# Respiratory impedance is correlated with morphological changes in the lungs on three-dimensional CT in patients with COPD

**DOI:** 10.1038/srep41709

**Published:** 2017-02-08

**Authors:** Masato Karayama, Naoki Inui, Kazutaka Mori, Masato Kono, Hironao Hozumi, Yuzo Suzuki, Kazuki Furuhashi, Dai Hashimoto, Noriyuki Enomoto, Tomoyuki Fujisawa, Yutaro Nakamura, Hiroshi Watanabe, Takafumi Suda

**Affiliations:** 1 Second Division, Department of Internal Medicine, Hamamatsu University School of Medicine, 1-20-1 Handayama, Hamamatsu 431-3192, Japan; 2 Department of Clinical Oncology, Hamamatsu University School of Medicine, 1-20-1 Handayama, Hamamatsu 431-3192, Japan; 3 Department of Clinical Pharmacology and Therapeutics, Hamamatsu University School of Medicine, 1-20-1 Handayama, Hamamatsu 431-3192, Japan

## Abstract

The forced oscillation technique provides information concerning respiratory impedance, which comprises resistance and reactance of the respiratory system. However, its relationship with morphological changes of the lungs in chronic obstructive pulmonary disease (COPD) remains unclear. Respiratory impedance and spirometric data were evaluated in 98 patients with COPD and 49 reference subjects. Wall thickness (WT) and airway intraluminal area (Ai) of third- to sixth-generation bronchi, and percentage low-attenuation area with less than −950 HU (%LAA) of lungs were measured using three-dimensional computed tomography. COPD patients had higher respiratory impedance, decreased Ai, and increased %LAA compared with reference subjects. Indices of respiratory resistance and reactance and forced expiratory volume in 1 second (FEV_1_) were correlated with Ai, and the association between percent predicted FEV_1_ and Ai was predominant in distal bronchi. The difference in respiratory resistance between 5 Hz and 20 Hz (R5–R20) and FEV_1_/forced vital capacity ratio (FEV_1_/FVC) were correlated with WT. The %LAA was correlated with the FEV_1_/FVC ratio and respiratory reactance. Airway function measurements with the forced oscillation technique provide complementary information to spirometry in COPD.

Chronic obstructive pulmonary disease (COPD) is characterized by persistent airflow limitation caused by inhalation of cigarette smoke and/or other noxious particles^1^. Small airway disease and parenchymal destruction are the key pathologic characteristics of COPD, and lead to air trapping and airflow limitation^1^,^2^,^3^.

Measurement of pulmonary function based on spirometry is required for the diagnosis of COPD, and is the most reproducible and objective measurement of airflow limitation among several methods available^1^. However, spirometric measurement is not sufficient for comprehensive assessment of COPD. Guidelines set by the Global Initiative for Chronic Obstructive Lung Disease (GOLD) emphasize the importance of multifaceted assessment for COPD in addition to spirometric assessment^1^.

Computed tomography (CT) of the chest provides information about pathologic changes in COPD. Studies have demonstrated that airway narrowing and thickening of airway walls on cross-sectional CT of the chest are associated with airflow limitation as assessed by spirometry in COPD patients^3^,^4^,^5^,^6^. Full assessment of airways by conventional CT is difficult because of the complicated structure with the branching framework of the lungs. However, three-dimensional (3D)-CT can be used to provide more detailed information of the airways and their correlations with physiologic function^7^,^8^,^9^,^10^,^11^.

The forced oscillation technique (FOT) can provide dynamic information regarding respiratory impedance (which comprises the resistance and reactance of the respiratory system)^12^,^13^,^14^. In patients with COPD, respiratory impedance measured by the FOT has demonstrated good correlation with spirometric data^15^,^16^,^17^,^18^,^19^ as well as with symptoms^20^,^21^. Moreover, respiratory impedance can be used to detect subtle changes in airway function in a manner that is even more sensitive than that seen for conventional spirometory^21^,^22^,^23^,^24^,^25^.

However, how and whether structural changes in the lung parenchyma and airways in COPD correlate with respiratory impedance is not known. Respiratory resistance at 5 Hz (R5) and 20 Hz (R20) are supposed to represent total airway resistance and large-airway resistance, respectively^12^,^14^. Respiratory reactance is considered to reflect the dynamic elastance and inertia of the respiratory system^12^,^14^. However, these interpretations are based not on direct measurement of the lung structure but instead are derived from a physical model or circumstantial evidence from studies that have compared respiratory impedance with other conventional physiologic tests (including spirometry)^12^,^14^,^26^. The association between respiratory impedance and imaging changes in the lungs could aid understanding of COPD pathology.

The aim of the present study was to evaluate potential correlations between respiratory impedance (assessed by the FOT) and airway structures (assessed by 3D-CT) in patients with COPD.

## Results

### Patient characteristics

Ninety-eight patients with COPD and 49 reference subjects were enrolled (Table 1). Patients with COPD had a median age of 73 years and 92.9% were male. All COPD patients had a history of smoking (median pack-year of 47). The median (range) value for percentage predicted forced expiratory volume in one second (%predicted FEV_1_) for COPD patients was 64.1 (22.9–114.9)% (Table 2). The percentage of GOLD stages I, II, III and IV were 20.4, 53.1, 19.4, and 7.1, respectively. Sixty-seven (68.4%) patients received a long-acting muscarinic antagonist, 76 (77.6%) received a long-acting beta-2 agonist, and 26 subjects (26.5%) received an inhaled corticosteroid. Twenty-eight (28.6%) patients received single inhaler therapy, 63 (64.3%) received double inhaler therapy, and 5 (5.1%) patients received triple inhaler therapy. Reference subjects comprised 14 never-smokers (28.6%) and 35 smokers (71.4%, median 40 pack-year, range 12–120) with normal spirometry. Reference subjects demonstrated a significantly younger age (a median of 67 years; range, 53–83), higher proportion of females (n = 12, 24.5%), and lower proportion of smokers, compared with patients with COPD.

### Correlation between 3D-CT of lungs and physiologic tests

Among 98 patients with COPD, Spearman rank correlation test was performed (Tables 3,4 and 5). The airway inner luminal area (Ai; mm^2^) demonstrated significant correlations with %predicted forced vital capacity (FVC), %predicted FEV_1_, FEV_1_/FVC ratio, and %predicted maximum mid-expiratory flow rate (MMF, Table 3). Among spirometric measurements, %predicted FEV_1_ had relatively stronger correlation with the Ai. Correlation coefficients between %predicted FEV_1_ and Ai tended to increase gradually towards the distal bronchi. Statistical analysis using Meng-Rosenthal-Rubin method showed that correlation coefficients were significantly different between sixth-generation and third-generation bronchi (*p* = 0.043), and between sixth-generation and fourth-generation bronchi (*p* = 0.025) (Supplementary Table S1). As for respiratory impedance, the Ai demonstrated significant inverse correlations with R5, R20, the difference between R5 and R20 (R5-R20), resonant frequency (Fres), and low-frequency reactance area (ALX), and significant correlation with respiratory reactance at 5 Hz (X5).

Wall thickness (WT; mm) demonstrated inverse correlations with FEV_1_/FVC ratio and %predicted MMF (Table 4). As for respiratory impedance, WT demonstrated positive correlations with R5, R5-R20, Fres, and ALX. There were no correlations between Ai and WT within same generation (Supplementary Table S2). To exclude interdependence between Ai and WT, we performed partial correlation analyses, which demonstrated that Ai and WT were independently correlated with spirometric data and respiratory impedance (Supplementary Tables S3 and 4).

Percentage low-attenuation area (%LAA) demonstrated significant inverse correlation with the FEV_1_/FVC ratio, %predicted FEV_1_, and %predicted MMF (Table 5). As for respiratory impedance, the %LAA demonstrated significant correlation with indices of respiratory reactance (X5, Fres, and ALX), whereas correlation with indices of respiratory resistance was not observed.

In contrast, Ai and WT showed no significant correlations with physiologic parameters in 49 reference subjects (Supplementary Table S5).

### Propensity score-matched analysis of physiologic tests and 3D-CT of lungs between patients with COPD and reference subjects

Next, we compared physiologic tests and 3D-CT of lungs between patients with COPD and reference subjects. Because our reference subjects demonstrated a significantly younger age, higher proportion of females, and lower proportion of smokers, compared with patients with COPD (Table 1), we used propensity score matching. After propensity matching with age, sex, and smoking status as co-variables, the two groups had comparable characteristics, except that patients with COPD had significantly lower %predicted FEV_1_, FEV_1_/FVC ratio, and %predicted MMF (Table 2). Patients with COPD had significantly higher R5, R20, R5–R20, Fres, and ALX, and significantly lower X5, compared with reference subjects (Table 6). Regarding 3D-CT, patients with COPD had significantly decreased Ai throughout the third- to sixth-generation bronchi, and significantly higher %LAA (Table 7). There was no significant difference in WT between the two groups.

## Discussion

In the present study, 3D-CT analyses of lungs revealed that respiratory impedance was correlated with airway narrowing, thickening of airway walls, and emphysema in patients with COPD. Airway narrowing was correlated with respiratory resistance and reactance. Among spirometric parameters, %predicted FEV_1_ showed a significant correlation with airway narrowing, which indicated a tendency of a gradual increase towards distal bronchi. The FEV_1_/FVC ratio, R5–R20, and respiratory reactance demonstrated correlations with the WT. Increased %LAA was correlated with the FEV_1_/FVC ratio and indices of respiratory reactance, but not with indices of respiratory resistance. The respiratory impedance as well as spirometric data may reflect different components of pathogenic changes in COPD, and could be used as complementary approaches for COPD assessment.

Respiratory resistance is considered to reflect the forward pressure of conducting airways^12^,^27^, and so is interpreted as an index of airway caliber^14^. The correlation between respiratory resistance and airway narrowing observed in the present study validates this theoretical model. Moreover, serial assessment of airways provided new insights regarding interpretation of respiratory resistance according to the proximal–distal axis. High-frequency waves reflect back from large airways and low-frequency waves travel deep into the lungs^12^. Therefore, it has been hypothesized that R5 denotes total airway resistance and R20 denotes central airways^12^,^14^. In the present study, R5 and R20 demonstrated almost similar correlations with airway narrowing in third- to sixth-generation bronchi assessed by 3D-CT.

Airway narrowing was also correlated with respiratory reactance. Usually, respiratory reactance reflects the elastic and inertial properties of the respiratory system^12^,^14^,^27^. However, if flow limitation is present, oscillatory signals cannot pass through flow-limiting segments (“choke points”) and reach alveoli, thereby eliciting marked reduction in apparent respiratory compliance and a decrease in respiratory reactance^26^. Therefore, respiratory reactance is considered to be a marker of flow limitation^16^,^17^,^28^. The correlation between respiratory reactance and airway narrowing observed in the present study may reflect flow limitation caused by obstruction of peripheral airways. It has been proposed that respiratory reactance reflects small airway function^16^,^17^,^29^, so detailed information about more distal airways may be necessary for further understanding of respiratory impedance.

In the present study, thickening of airway walls was correlated with respiratory impedance. The correlations between WT and impedance were independent of Ai, and vice versa, according to the partial correlation analysis. Peribronchial fibrosis and consequent remodeling of airway tissue associated with COPD progression reduces airway compliance, which may result in a decrease in respiratory reactance^30^,^31^. The correlation between thickening of airways walls and respiratory reactance observed in the present study was compatible with this hypothesis. Airway fibrosis may also affect tissue resistance (a component of respiratory resistance^14^) and contribute to the correlation between thickening of airway walls and respiratory resistance. R5–R20 is considered to reflect non-uniform distribution of ventilation^12^,^14^, and we cannot explain the relationship between thickening of airway walls and increase in R5-R20. Thickening of airway walls could represent COPD progression, correlate with ventilation “unevenness,” and be related indirectly with R5–R20 as a potential confounding factor. The relationship between the FEV_1_/FVC ratio and thickening of airway walls is also unclear, but these physiologic parameters could aid prediction of airway remodeling in COPD.

The increased %LAA was correlated with indices of respiratory reactance. Emphysema is one of the key pathologic characteristics of COPD, which reflects parenchymal destruction. Emphysema displays a patchy distribution and causes heterogeneous airway collapse, which may be associated with respiratory reactance denoting non-uniform ventilation^16^,^29^.

The present study had several limitations. First, respiratory impedance cannot be explained entirely by airway structure alone. Respiratory resistance includes tissue resistance and chest-wall resistance (though airway resistance is the major part^14^). Secretion within the airway luminal area (a key component of airway obstruction in COPD^12^) could not be evaluated in our study. These potentially influential factors (other than structural features of airways) should be considered for interpretation of respiratory impedance. Second, the limited resolution of CT could not aid evaluation of peripheral airways more distal than sixth-generation bronchi. Major sites of airway obstruction in COPD are small airways <2 mm in diameter. We evaluated third- to sixth-generation bronchi, but more distal airways (e.g. terminal bronchioles) are also important in COPD^3^. Third, static CT images obtained at full-inspiration could not account fully for dynamic changes in the lungs during tidal breathing. Airway caliber collapses in the expiratory phase because of low elastic recoil pressure due to parenchymal destruction (emphysema)^32^,^33^, which is thought to be associated with within-breath difference in respiratory impedance^17^,^31^,^34^. Time-series analyses of airways could provide further insights into respiratory impedance. Finally, our reference subjects demonstrated a significantly younger age and higher proportion of females. Although the proportion of smoker in the reference subjects was lower than that in COPD patients, relatively few were non-smokers and the majority was smoker, all which may have affected the results. Comparison of patients with COPD and non-smoking reference subjects (only 14 subjects) showed that COPD patients had thicker walls, narrower airways, and higher respiratory impedance, although some of the differences were not significant. In the present study, we used propensity-score matching analysis to minimize the potential influence of heterogeneity among the reference group. After propensity matching with age, sex, and smoking status as co-variables, the two groups had comparable characteristics.

Respiratory impedance was correlated with airway narrowing, thickening of airway walls, and emphysema in patients with COPD. Complementary use of FOT and spirometry may be useful for evaluation of pathogenic changes in COPD.

## Methods

### Study design

This was a prospective observational study conducted in accordance with the ethical standards described in the Declaration of Helsinki. The study protocol was approved by the Institutional Review Board of Hamamatsu University School of Medicine (Hamamatsu, Japan). Each patient provided written informed consent to be included in the study. The study was registered with the University Hospital Medical Information Network Clinical Trial Registry (identification code: 000013541).

### Patient eligibility

Patients who satisfied the definition of COPD set by GOLD^1^ were enrolled. Exclusion criteria were: the requirement of treatment change, respiratory-tract infection, and COPD exacerbation 4 weeks before study commencement; long-term oxygen therapy; bronchial asthma; diffuse lung diseases; neuromuscular diseases; congenital anomalies of the bronchial tree; history of thoracic surgery. Subjects without COPD were enrolled as a reference population and were required to: (i) have normal pulmonary function as assessed by spirometry; (ii) not meet the exclusion criteria described above. Propensity score-matched analysis was performed to minimize biases between patients with COPD and reference subjects in one-to-two matching using age, sex, and smoking status as co-variables.

### Multi-detector-row computed tomography (MDCT)

MDCT was done in the supine position at full-inspiration breath-hold using a 64-slice MDCT scanner (Aquilion-64; Toshiba Medical Systems, Tokyo, Japan). Scanning parameters were: collimation, 64 × 0.5 mm; tube voltage, 120 kV; tube current, 200 mA; rotation time, 0.5 s; pitch, 0.83. These parameters were varied by the scanner system to obtain the optimum radiation dose and image quality. Images were reconstructed using a standard reconstruction algorithm (FC50) for the lung using a slice thickness of 0.5 mm and reconstruction interval of 0.5 mm.

### Three-dimensional (3D) CT analysis of lungs

**Figure 1:**
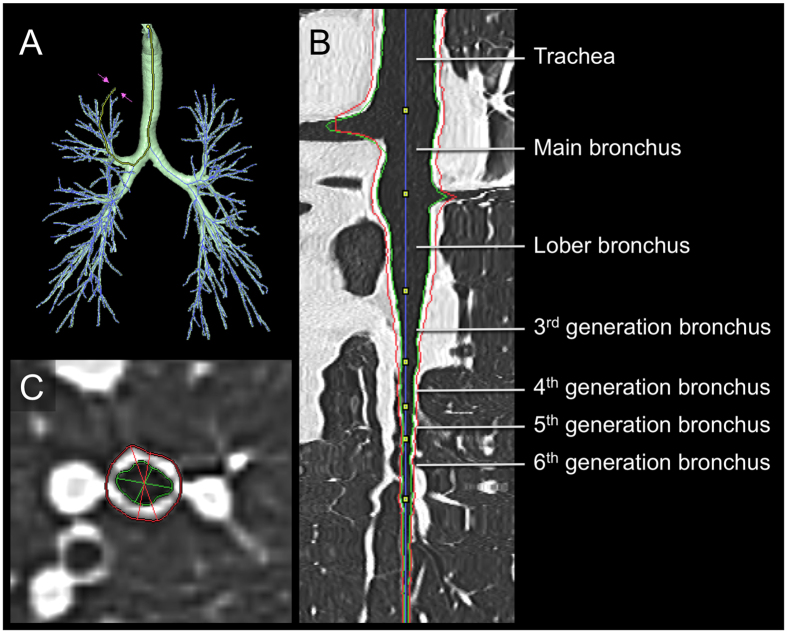
Three-dimensional CT analysis of the airway. (**A**) The image of the airway tree reconstructed by three-dimensional computed tomography. (**B**) The longitudinal section of the reformatted airway in the multiplanar reconstruction image. (**C**) Measurement of the airway in the short axis image. Red and green lines indicate the outer and inner edge of the airway, respectively.

Using a reconstructed 3D-CT method, we obtained images of airways with image-analyzing software (SYNAPSE VINCENT; Fuji Film, Tokyo, Japan). The bronchial pathway was reconstructed automatically into multiplanar reconstruction images with a window width of 1600 HU and a window level of –600 HU (Fig. 1). Measurements of airways were done in four ways according to methods reported previously^11^. First, six segmental bronchi (B1, B2, B3, B8, B9, and B10) in the right lung were selected. Middle-lobe bronchi as well as B6 and B7 were excluded from analyses because they are readily associated with atelectasis, airway collapse and mucus hypersecretion. The left lung was also excluded from analyses to avoid artifacts due to transmitted cardiac motion. Second, in each selected bronchus, four levels of the airways (third- (segmental), fourth- (sub-segmental), fifth-, and sixth-generation bronchi) were identified by tracing airway trees peripherally on the same trunk of each segmental bronchus. Third, in the midpoint of each level of airway, WT and Ai perpendicular to the long axis of the airway were computed automatically (Fig. 1). Airway center line was constructed by using minimum spanning tree, and inner and outer airway contours were extracted by using graph cuts method. WT was calculated as the mean distance of the outer to inner edge of the airway assuming that it is a “true” circle. The inner and outer airway contours were extracted automatically by the image-analysis software. If an error occurred during automatic extraction of airway contours, then it was corrected by manual means. Fourth, six images were evaluated at each level of the bronchus, and the results for bronchi from each generation were expressed separately as the means of six airways. The %LAA, defined by the percentage of area below −950 HU in total lung area, was calculated using SYNAPSE VINCENT.

### Measurement of spirometry and respiratory impedance

Spirometry and the FOT were performed at the same day of the chest CT scans. The FOT was performed before spirometry to avoid influences of forced breathing. Short-acting β_2_-agonists were not withheld for at least 12 hours before these tests. Autospirometer System 7 (Minato Medical Science Co., Ltd., Osaka, Japan) was used to measure spritometry according to the standards of the Japanese Respiratory society^35^. Respiratory impedance was measured using a commercial FOT device (Most-Graph 01; Chest MI, Tokyo, Japan) according to standard recommendations as reported previously^17^,^27^. Briefly, impulse oscillatory signals generated by a loudspeaker at intervals of 0.25 s were applied to the respiratory system through the mouthpiece during tidal breathing at rest. Mouth pressure and flow signals were measured and calculated to obtain the resistance and reactance properties against oscillatory frequencies from 4–36 Hz. We evaluated R5, R20, R5–R20, X5, Fres (resonant frequency; where the reactance crosses zero and the elastic and inertial forces are equal in magnitude and opposite), and ALX (low-frequency reactance area; the integral of reactance from 5 Hz to Fres). Each index was expressed as a mean value at whole-breath phase.

### Statistical analyses

The Wilcoxon signed rank test was used for continuous variables and Fisher’s exact test for categorical groups. Correlation between continuous variables was undertaken using the Spearman rank correlation coefficient. Partial correlation analyses were performed to exclude the potential confounding between Ai and WT. Difference between correlation coefficients was evaluated by Meng-Rosenthal-Rubin method. Propensity score-matched analysis was performed using EZR (Saitama Medical Center, Jichi Medical University, Saitama, Japan), which is a graphical user interface for R (The R Foundation for Statistical Computing, Vienna, Austria). Data are the median (range) unless indicated otherwise. Statistical tests were two-sided, and *p* < 0.05 was considered significant. Values were analyzed using JMP v9.0.0 (SAS Institute Japan, Tokyo, Japan), except for partial correlation analyses and Meng-Rosenthal-Rubin method using R version 3.2.4 (The R Foundation for Statistical Computing, Vienna, Austria, 2016).

## Additional Information

**How to cite this article**: Karayama, M. *et al*. Respiratory impedance is correlated with morphological changes in the lungs on three-dimensional CT in patients with COPD. *Sci. Rep.* **7**, 41709; doi: 10.1038/srep41709 (2017).

**Publisher's note:** Springer Nature remains neutral with regard to jurisdictional claims in published maps and institutional affiliations.

## Supplementary Material

Supplementary Tables

## Figures and Tables

**Table 1:** Patient characteristics.

	All population			Matched population^†^		
	COPD (n = 98)	Reference (n = 49)	*p*-value*	COPD (n = 70)	Reference (n = 35)	*p*-value*
Age, years	73 (49–89)	67 (53–83)	0.002	70 (49–84)	67 (48–83)	0.221
Sex: Male	91 (92.9)	37 (75.5)	0.007^¶^	67 (95.7)	34 (97.1)	1.000^¶^
Smoker	98 (100)	35 (71.4)	<0.001^¶^	70 (100)	35 (100)	N.E.
Pack-year	47 (10–250)	30 (0–120)	<0.001	47 (10–250)	40 (12–120)	0.654
Height, m	1.64 (1.42–1.86)	1.61 (1.44–1.76)	0.245	1.64 (1.49–1.86)	1.65 (1.47–1.76)	0.878
Weight, kg	59.3 (37.4–93.6)	57.1 (33.3–77.9)	0.785	60.8 (37.4–93.6)	63.3 (34.9–77.9)	0.370
BMI, kg/m^2^	22.7 (15.4–33.2)	22.5 (14.4–28.7)	0.455	22.7 (15.4–33.2)	22.8 (14.7–28.6)	0.229
GOLD stage						
I/II/III/IV	20 (20.4)/52 (53.1)/19 (19.4)/7 (7.1)			14 (20.0)/40 (57.1)/11 (15.7)/5 (7.1)		
Treatment						
LAMA	19 (19.4)			13 (18.6)		
LABA	9 (9.2)			7 (10.0)		
LAMA/LABA	42 (42.9)			27 (38.6)		
ICS/LABA	20 (20.4)			0 (0)		
ICS/LAMA	1 (1.0)			1 (1.4)		
ICS/LAMA/LABA	5 (5.1)			3 (4.3)		

Data are expressed as number (%) or median (range). ^†^Propensity score-matched population with age, sex, and smoking status. COPD, chronic obstructive pulmonary disease; BMI, body mass index; FVC, forced vital capacity; FEV_1_, forced expiratory volume in 1 second; MMF, maximum mid-expiratory flow rate; GOLD, Global Initiative for Asthma. LABA, long-acting beta 2 agonist; LAMA, long-acting muscarinic antagonist; ICS, inhaled corticosteroid; N.E., not estimated. *Wilcoxon signed-rank test unless otherwise indicated. ^¶^Fisher’s exact test.

**Table 2:** Spirometric data.

	All population			Matched population^†^		
	COPD (n = 98)	Reference (n = 49)	*p*-value*	COPD (n = 70)	Reference (n = 35)	*p*-value*
FVC (L)	3.00 (1.14–5.43)	3.24 (1.80–4.54)	0.189	3.11 (1.14–5.43)	3.36 (2.19–4.54)	0.050
FVC, % predicted	93.1 (54.8–134.9)	99.8 (80.3–174.7)	0.009	92.6 (57.5–129.7)	99.2 (80.3–121.4)	0.034
FEV_1_ (L)	1.66 (0.45–3.39)	2.40 (1.68–3.50)	<0.001	1.82 (0.54–3.39)	2.65 (1.69–3.50)	<0.001
FEV_1_, % predicted	64.1 (22.9–114.9)	97.0 (80.0–163.8)	<0.001	65.4 (23.3–112.7)	95.4 (80.0–110.6)	<0.001
FEV_1_/FVC (%)	55.9 (26.6–69.9)	77.7 (70.0–95.8)	<0.001	60.0 (26.6–69.9)	76.7 (70.0–95.8)	<0.001
MMF (L/S)	0.66 (0.17–7.10)	2.08 (1.16–8.59)	<0.001	0.78 (0.18–7.10)	2.2 (1.16–7.41)	<0.001
MMF, %predicted	23.1 (6.3–83.1)	76.0 (44.4–135.9)	<0.001	28.3 (6.3–83.1)	70.2 (44.4–135.9)	<0.001

Data are expressed as median (range). ^†^Propensity score-matched population with age, sex, and smoking status. Abbreviations are as described in Table 1. *Wilcoxon signed-rank test.

**Table 3:** Correlations between airway luminal areas and physiological function tests in patients with COPD.

	3rd-generation bronchi		4th-generation bronchi		5th-generation bronchi		6th-generation bronchi	
	r	*p*-value	r	*p*-value	r	*p*-value	r	*p*-value
Spirometry								
FVC, %predicted	0.349	<0.001	0.291	0.005	0.385	<0.001	0.418	<0.001
FEV_1_, %predicted	0.375	<0.001	0.392	<0.001	0.474	<0.001	0.548	<0.001
FEV_1_/FVC ratio	0.235	0.024	0.322	0.002	0.339	<0.001	0.415	<0.001
MMF, %predicted	0.321	0.002	0.368	<0.001	0.423	<0.001	0.497	<0.001
Respiratory impedance, whole-breath								
R5	−0.508	<0.001	−0.492	<0.001	−0.435	<0.001	−0.435	<0.001
R20	−0.460	<0.001	−0.486	<0.001	−0.422	<0.001	−0.421	<0.001
R5-R20	−0.400	<0.001	−0.330	0.001	−0.324	0.002	−0.318	0.002
X5	0.394	<0.001	0.317	0.002	0.304	0.003	0.364	<0.001
Fres	−0.472	<0.001	−0.392	<0.001	−0.361	<0.001	−0.384	<0.001
ALX	−0.431	<0.001	−0.362	<0.001	−0.335	0.001	−0.372	<0.001

Data are expressed as Spearman rank correlation coefficient and *p*-value. R5, respiratory resistance at 5 Hz; R 20, respiratory resistance at 20 Hz: X5, respiratory reactance at 5 Hz; Fres, resonant frequency; ALX, low-frequency reactance area. The other abbreviations are as described in Table 1.

**Table 4:** Correlations between airway wall thickness and physiological function tests in patients with COPD.

	3rd-generation bronchi		4th-generation bronchi		5th-generation bronchi		6th-generation bronchi	
	r	*p*-value	r	*p*-value	r	*p*-value	r	*p*-value
Spirometry								
FVC, %predicted	−0.019	0.853	0.020	0.847	0.053	0.617	0.055	0.598
FEV_1_, %predicted	−0.172	0.098	−0.189	0.070	−0.200	0.054	−0.187	0.073
FEV_1_/FVC ratio	−0.269	0.009	−0.320	0.002	−0.326	0.001	−0.323	0.002
MMF, %predicted	−0.182	0.081	−0.221	0.033	−0.225	0.030	−0.229	0.027
Respiratory impedance, whole-breath								
R5	0.257	0.013	0.294	0.004	0.150	0.150	0.093	0.375
R20	0.176	0.092	0.212	0.041	0.056	0.595	−0.015	0.885
R5-R20	0.358	<0.001	0.384	<0.001	0.313	0.002	0.276	0.007
X5	−0.220	0.034	−0.218	0.036	−0.140	0.180	−0.115	0.272
Fres	0.264	0.010	0.308	0.003	0.205	0.048	0.206	0.048
ALX	0.275	0.008	0.301	0.003	0.181	0.082	0.170	0.103

Data are expressed as Spearman rank correlation coefficient and *p*-value. The other abbreviations are as described in Tables 1 and 3.

**Table 5:** Correlations between percentage of low attenuation area and physiological function tests in patients with COPD.

	r	*p*-value
Spirometry		
FVC, %predicted	−0.153	0.133
FEV_1_, %predicted	−0.464	<0.001
FEV_1_/FVC ratio	−0.580	<0.001
MMF, %predicted	−0.453	<0.001
Respiratory impedance, whole-breath		
R5	0.082	0.421
R20	0.026	0.802
R5-R20	0.182	0.073
X5	−0.276	0.006
Fres	0.304	0.002
ALX	0.255	0.011

Data are expressed as Spearman rank correlation coefficient and *p*-value. %LAA, percentage of low attenuation area <−950 HU; The other abbreviations are as described in Tables 1 and 3.

**Table 6:** Respiratory impedance at whole-breath.

	All population			Matched population^†^		
	COPD (n = 98)	Reference (n = 49)	*p*-value*	COPD (n = 70)	Reference (n = 35)	*p*-value*
R5 (cmH_2_O/L/s)	3.17 (0.52–6.38)	2.46 (0.97–4.96)	<0.001	3.11 (0.52–6.38)	1.86 (0.97–4.96)	<0.001
R20 (cmH_2_O/L/s)	2.51 (0.53–4.55)	2.11 (1.02–4.60)	0.004	2.45 (0.53–4.55)	1.83 (1.02–4.60)	0.002
R5-R20 (cmH_2_O/L/s)	0.66 (−0.63–2.14)	0.37 (−0.52–1.86)	<0.001	0.53 (−0.63–1.88)	0.23 (−0.52–1.86)	0.001
X5 (cmH_2_O/L/s)	−0.72 (−5.35–0.37)	−0.33 (−2.18–0.24)	<0.001	−0.59 (−5.35–0.37)	−0.31 (−2.18–0.24)	0.005
Fres (Hz)	11.27 (4.00–30.17)	7.38 (4.16–16.04)	<0.001	10.71 (4.00–30.17)	7.31 (4.16–16.04)	<0.001
ALX (cmH_2_O/L/s × Hz)	3.76 (0–51.26)	1.14 (0.02–14.59)	<0.001	2.66 (0–51.26)	1.06 (0.02–14.59)	0.002

Data are expressed as median (range). ^†^Propensity score-matched population with age, sex, and smoking status. The other abbreviations are as described in Table 3. *Wilcoxon signed-rank test.

**Table 7:** Three-dimensional CT imaging of the lungs.

	All population			Matched population^†^		
	COPD (n = 98)	Reference (n = 49)	*p*-value*	COPD (n = 70)	Reference (n = 35)	*p*-value*
Ai (mm^2^)						
3rd-generation bronchi	35.9 (18.2–64.0)	41.2 (18.9–76.8)	0.007	35.7 (18.2–64.0)	41.7 (23.2–76.8)	0.002
4th-generation bronchi	9.8 (3.7–20.4)	11.1 (5.5–25.1)	0.007	9.8 (4.6–18.2)	11.5 (6.1–25.1)	0.005
5th-generation bronchi	5.8 (2.4–11.7)	7.1 (3.0–12.0)	<0.001	5.8 (2.6–11.6)	7.1 (3.8–12.0)	<0.001
6th-generation bronchi	3.3 (0.9–7.7)	4.3 (1.7–7.9)	<0.001	3.2 (0.9–6.9)	4.2 (2.3–7.9)	<0.001
WT (mm)						
3rd-generation bronchi	1.6 (1.2–2.0)	1.5 (1.3–1.9)	0.005	1.6 (1.2–1.9)	1.5 (1.3–1.8)	0.268
4th-generation bronchi	1.4 (1.1–1.8)	1.3 (1.0–1.5)	0.010	1.4 (1.1–1.6)	1.3 (1.2–1.6)	0.126
5th-generation bronchi	1.2 (0.9–1.5)	1.2 (1.0–1.4)	0.085	1.2 (0.9–1.5)	1.2 (1.1–1.4)	0.407
6th-generation bronchi	1.1 (0.9–1.3)	1.1 (0.9–1.3)	0.194	1.1 (0.9–1.3)	1.1 (0.9–1.3)	0.679
%LAA	41.4 (17.1–64.2)	29.6 (11.2–51.6)	<0.001	40.9 (20.7–64.2)	31.5 (14.0–51.6)	<0.001

Data are expressed as median (range). ^†^Propensity score-matched population with age, sex, and smoking status. COPD, chronic obstructive pulmonary disease; Ai, airway inner luminal area; WT, airway wall thickness; %LAA, percentage of low attenuation area <−950 HU. *Wilcoxon signed-rank test.

## References

[b1] The Global Strategy for the Diagnosis, Management and Prevention of COPD, Global Initiative for Chronic Obstructive Lung Disease (GOLD) 2016 (Date of access: 30/07/2016) Available from: http://goldcopd.org/ (2016).

[b2] Hogg J. C. . The Nature of small-airway obstruction in chronic obstructive pulmonary disease. N Engl J Med 350, 2654–2653 (2004). 15215480 10.1056/NEJMoa032158

[b3] McDonough J. E. . Small-airway obstruction and emphysema in chronic obstructive pulmonary disease. N Engl J Med 365, 1567–1575 (2011). 22029978 10.1056/NEJMoa1106955 PMC3238466

[b4] Nakano Y. . Computed tomographic measurements of airway dimensions and emphysema in smokers correlation with lung function. Am. J. Respir. Crit. Care Med. 162, 1102–1108 (2000). 10988137 10.1164/ajrccm.162.3.9907120

[b5] Berger P. . Airway wall thickness in cigarette smokers: quantitative thin-section CT assessment. Radiology 235, 1055–1064 (2005). 15833982 10.1148/radiol.2353040121

[b6] Aziz Z. A. . Functional impairment in emphysema: Contribution of airway abnormalities and distribution of parenchymal disease. Am. J. Roentgenol. 185, 1509–1515 (2005). 16304005 10.2214/AJR.04.1578

[b7] Hasegawa M. . Airflow limitation and airway dimensions in chronic obstructive pulmonary disease. Am. J. Respir. Crit. Care Med. 173, 1309–1315 (2006). 16556695 10.1164/rccm.200601-037OC

[b8] Coxson H. O. . Airway wall thickness assessed using computed tomography and optical coherence tomography. Am. J. Respir. Crit. Care Med. 177, 1201–1206 (2008). 18310475 10.1164/rccm.200712-1776OC PMC2408438

[b9] Coxson H. O. Quantitative computed tomography assessment of airway wall dimensions: current status and potential applications for phenotyping chronic obstructive pulmonary disease. Proc. Am. Thorac. Soc. 5, 940–945 (2008). 19056721 10.1513/pats.200806-057QC PMC2720108

[b10] Hasegawa M. . Relationship between improved airflow limitation and changes in airway calibre induced by inhaled anticholinergic agents in COPD. Thorax 64, 332–338 (2009). 19074932 10.1136/thx.2008.103671

[b11] Yasui H. . Multidetector-row computed tomography assessment of adding budesonide/formoterol to tiotropium in patients with chronic obstructive pulmonary disease. Pulm. Pharmacol. Ther. 26, 336–41 (2013). 23340058 10.1016/j.pupt.2013.01.005

[b12] Bickel S., Popler J., Lesnick B. & Eid N. Impulse oscillometry: Interpretation and practical applications. Chest 146, 841–847 (2014). 25180727 10.1378/chest.13-1875

[b13] Oostveen E. . The forced oscillation technique in clinical practice: methodology, recommendations and future developments. Eur. Respir. J. 22, 1026–1041 (2003). 14680096 10.1183/09031936.03.00089403

[b14] Shirai T. & Kurosawa H. Clinical application of the forced oscillation technique. Intern. Med. 55, 559–66 (2016). 26984069 10.2169/internalmedicine.55.5876

[b15] Timmins S. C. . Day-to-day variability of oscillatory impedance and spirometry in asthma and COPD. Respir. Physiol. Neurobiol. 185, 416–424 (2013). 22960661 10.1016/j.resp.2012.08.017

[b16] Mikamo M. . Predictors of expiratory flow limitation measured by forced oscillation technique in COPD. BMC Pulm. Med. 14, 23 (2014). 24552475 10.1186/1471-2466-14-23 PMC3936701

[b17] Mori K. . Colored 3-dimensional analyses of respiratory resistance and reactance in COPD and asthma. COPD 8, 456–463 (2011). 22149407 10.3109/15412555.2011.626818

[b18] Pisi R. . Small airway dysfunction and flow and volume bronchodilator responsiveness in patients with chronic obstructive pulmonary disease. Int. J. Chron. Obstruct. Pulmon. Dis. 10, 1191–7 (2015). 26150710 10.2147/COPD.S82509 PMC4480584

[b19] Kolsum U. . Impulse oscillometry in COPD: Identification of measurements related to airway obstruction, airway conductance and lung volumes. Respir. Med. 103, 136–143 (2009). 18760576 10.1016/j.rmed.2008.07.014

[b20] Jetmalani K. . Expiratory flow limitation relates to symptoms during COPD exacerbations requiring hospital admission. Int. J. Chron. Obstruct. Pulmon. Dis. 10, 939–945 (2015). 25999709 10.2147/COPD.S78332 PMC4437522

[b21] Timmins S. C. . Changes in oscillatory impedance and nitrogen washout with combination fluticasone/salmeterol therapy in COPD. Respir. Med. 108, 344–350 (2014). 24144670 10.1016/j.rmed.2013.10.004

[b22] Shinke H. . Visualized changes in respiratory resistance and reactance along a time axis in smokers: A cross-sectional study. Respir. Investig. 51, 166–174 (2013). 10.1016/j.resinv.2013.02.006 23978643

[b23] Frantz S. . Impulse oscillometry may be of value in detecting early manifestations of COPD. Respir. Med. 106, 1116–1123 (2012). 22613172 10.1016/j.rmed.2012.04.010

[b24] Abe T. . Effects of inhaled tiotropium plus transdermal tulobuterol versus tiotropium alone on impulse oscillation system (IOS)-assessed measures of peripheral airway resistance and reactance, lung function and quality of life in patients with COPD: A randomized crossover study. Pulm. Pharmacol. Ther. 24, 617–624 (2011). 21689775 10.1016/j.pupt.2011.06.002

[b25] Oppenheimer B. W. . Distal airway function in symptomatic subjects with normal spirometry following world trade center dust exposure. Chest 132, 1275–1282 (2007). 17890470 10.1378/chest.07-0913

[b26] Dellacà R. L. . Detection of expiratory flow limitation in COPD using the forced oscillation technique. Eur. Respir. J. 23, 232–240 (2004). 14979497 10.1183/09031936.04.00046804

[b27] Oostveen E. . The forced oscillation technique in clinical practice: methodology, recommendations and future developments. Eur. Respir. J. 22, 1026–1041 (2003). 14680096 10.1183/09031936.03.00089403

[b28] Akita T. . Association of the forced oscillation technique with negative expiratory pressure in COPD. Respir. Physiol. Neurobiol. 220, 62–68 (2016). 26369446 10.1016/j.resp.2015.09.002

[b29] Bhatawadekar S. A., Leary D. & Maksym G. N. Modelling resistance and reactance with heterogeneous airway narrowing in mild to severe asthma. Can. J. Physiol. Pharmacol. 93, 207–214 (2015). 25730711 10.1139/cjpp-2014-0436

[b30] Silva K. K. D., Lopes A. J., Jansen J. M. & Melo P. L. de. Total inspiratory and expiratory impedance in patients with severe chronic obstructive pulmonary disease. Clinics 66, 2085–2091 (2011). 22189734 10.1590/S1807-59322011001200014 PMC3226604

[b31] Silva K., Faria A., Lopes A. & Melo P. Within-breath respiratory impedance and airway obstruction in patients with chronic obstructive pulmonary disease. Clinics 70, 461–469 (2015). 26222814 10.6061/clinics/2015(07)01 PMC4496751

[b32] Kurosawa H. & Kohzuki M. Images in Clinical Medicine. Dynamic airway narrowing. N. Engl. J. Med. 350, 1036 (2004). 14999115 10.1056/NEJMicm030626

[b33] Hasegawa K. . Emphysema and airway disease affect within-breath changes in respiratory resistance in COPD patients. Respirology 20, 775–781 (2015). 25824559 10.1111/resp.12535

[b34] Yamauchi Y., Kohyama T., Jo T. & Nagase T. Dynamic change in respiratory resistance during inspiratory and expiratory phases of tidal breathing in patients with chronic obstructive pulmonary disease. Int. J. Chron. Obstruct. Pulmon. Dis. 7, 259–69 (2012). 22589578 10.2147/COPD.S30399 PMC3346211

[b35] Guidelines of Respiratory Function Tests: spirometry, flow-volume curve, and diffusion capacity of the lung. (ed. The Japanese Respiratory Society) 2–23 (Tokyo, 2004). 15565748

