# Supplementary material for: Respiratory impedance is correlated with morphological changes in the lungs on three-dimensional CT in patients with COPD
Source: Sci Rep. 2017 Feb 8;7:41709. doi: 10.1038/srep41709 (PMC5296866; doi:10.1038/srep41709)
Supplement: Supplementary Tables [file srep41709-s1.doc]

***Supplementary Information***

Respiratory impedance is correlated with morphological changes in the lungs on three-dimensional CT in patients with COPD

Masato Karayama^1,2^, Naoki Inui^1,3^*. Kazutaka Mori^1^, Masato Kono^1^, Hironao Hozumi^1^, Yuzo Suzuki^1^, Kazuki Furuhashi^1^, Dai Hashimoto^1^, Noriyuki Enomoto^1^, Tomoyuki Fujisawa^1^, Yutaro Nakamura^1^, Hiroshi Watanabe^3^, Takafumi Suda^1^

^1^Second Division, Department of Internal Medicine, Hamamatsu University School of Medicine, 1-20-1 Handayama, Hamamatsu 431-3192, Japan

^2^Department of Clinical Oncology, Hamamatsu University School of Medicine, 1-20-1 Handayama, Hamamatsu 431-3192, Japan

^3^Department of Clinical Pharmacology and Therapeutics, Hamamatsu University School of Medicine, 1-20-1 Handayama, Hamamatsu 431-3192, Japan

*Corresponding author

E-mail: [inui@hama-med.ac.jp](mailto:inui@hama-med.ac.jp) (NI)

Supplementary Table S1. Differences in the magnitude of correlations with airway inner luminal areas and physiological function tests among bronchial generations.

|  | 3rd – 4th | | 3rd – 5th | | 3th – 6th | | 4th – 5th | | 4th – 6th | | 5th – 6th | |
| --- | --- | --- | --- | --- | --- | --- | --- | --- | --- | --- | --- | --- |
|  | Δr | *p*-value | Δr | *p*-value | Δr | *p*-value | Δr | *p*-value | Δr | *p*-value | Δr | *p*-value |
| FVC, % | 0.057 | 0.318 | –0.036 | 0.632 | –0.069 | 0.445 | –0.093 | 0.080 | –0.126 | 0.093 | –0.033 | 0.565 |
| FEV_1_, % | –0.016 | 0.773 | –0.099 | 0.175 | –0.173 | 0.043 | –0.083 | 0.105 | –0.157 | 0.025 | –0.074 | 0.164 |
| FEV_1_/FVC | –0.088 | 0.131 | –0.105 | 0.180 | –0.181 | 0.052 | –0.017 | 0.756 | –0.093 | 0.214 | –0.076 | 0.188 |
| MMF, % | –0.047 | 0.408 | –0.102 | 0.175 | –0.175 | 0.048 | –0.055 | 0.291 | –0.128 | 0.074 | –0.074 | 0.184 |
| R5 | –0.016 | 0.761 | –0.073 | 0.303 | –0.073 | 0.388 | –0.057 | 0.255 | –0.058 | 0.414 | –0.001 | 0.989 |
| R20 | 0.026 | 0.628 | –0.038 | 0.596 | –0.040 | 0.649 | –0.064 | 0.203 | –0.065 | 0.358 | –0.001 | 0.980 |
| R5-R20 | –0.069 | 0.218 | –0.076 | 0.316 | –0.081 | 0.376 | –0.006 | 0.904 | –0.012 | 0.877 | –0.005 | 0.929 |
| X5 | 0.077 | 0.175 | 0.090 | 0.236 | 0.030 | 0.739 | 0.013 | 0.805 | –0.046 | 0.539 | –0.060 | 0.312 |
| Fres | –0.080 | 0.140 | –0.111 | 0.129 | –0.088 | 0.318 | –0.031 | 0.554 | –0.008 | 0.919 | 0.024 | 0.685 |
| ALX | –0.069 | 0.214 | –0.096 | 0.199 | –0.059 | 0.507 | –0.027 | 0.611 | 0.010 | 0.898 | 0.037 | 0.532 |

Data are expressed as difference in Spearman rank correlation coefficient (Δr) and *p*-value in Meng-Rosenthal-Rubin method. 3rd, third-generation bronchi; 4th, fourth-generation bronchi; 5th, fifth-generation bronchi; 6th, sixth-generation bronchi; FVC, %, percent predicted forced vital capacity; FEV_1_, %, percent predicted forced expiratory volume in 1 second; MMF, %, percent predicted maximum mid-expiratory flow rate; R5, respiratory resistance at 5 Hz; R 20, respiratory resistance at 20 Hz: X5, respiratory reactance at 5 Hz; Fres, resonant frequency; ALX, low-frequency reactance area.

Supplementary Table S2. Correlations between airway inner luminal area and wall thickness.

|  | Third-generation bronchi | | Fourth-generation  bronchi | | Fifth-generation  bronchi | | Sixth-generation  bronchi | |
| --- | --- | --- | --- | --- | --- | --- | --- | --- |
|  | r | *p*-value | r | *p*-value | r | *p*-value | r | *p*-value |
| Correlation between Ai and WT | -0.115 | 0.274 | -0.135 | 0.196 | -0.029 | 0.783 | 0.021 | 0.844 |

Data are expressed as Spearman rank correlation coefficient (r) and *p*-value. Ai, airway inner luminal area; WT, wall thickness. The other abbreviations are as described in Table S1.

Supplementary Table S3. Partial correlations between airway luminal areas and physiological function tests adjusted for airway wall thickness in patients with COPD.

|  | Third-generation bronchi | | Fourth-generation bronchi | | Fifth-generation bronchi | | Sixth-generation bronchi | |
| --- | --- | --- | --- | --- | --- | --- | --- | --- |
|  | r | *p*-value | r | *p*-value | r | *p*-value | r | *p*-value |
| Spirometry |  |  |  |  |  |  |  |  |
| FVC, %predicted | 0.349 | <0.001 | 0.297 | 0.003 | 0.387 | <0.001 | 0.417 | <0.001 |
| FEV_1_, %predicted | 0.364 | <0.001 | 0.376 | <0.001 | 0.478 | <0.001 | 0.562 | <0.001 |
| FEV_1_/FVC ratio | 0.213 | 0.036 | 0.297 | 0.003 | 0.349 | <0.001 | 0.446 | <0.001 |
| MMF, %predicted | 0.308 | 0.002 | 0.350 | <0.001 | 0.428 | <0.001 | 0.515 | <0.001 |
| Respiratory impedance, whole-breath | | | | | | | | |
| R5 | ­0.499 | <0.001 | ­0.478 | <0.001 | ­0.436 | <0.001 | ­0.439 | <0.001 |
| R20 | ­0.450 | <0.001 | ­0.472 | <0.001 | ­0.421 | <0.001 | ­0.421 | <0.001 |
| R5-R20 | ­0.387 | <0.001 | ­0.305 | 0.002 | ­0.332 | <0.001 | ­0.338 | <0.001 |
| X5 | 0.381 | <0.001 | 0.298 | 0.003 | 0.303 | 0.003 | 0.369 | <0.001 |
| Fres | ­0.461 | <0.001 | ­0.372 | <0.001 | ­0.363 | <0.001 | ­0.398 | <0.001 |
| ALX | ­0.419 | <0.001 | ­0.341 | <0.001 | ­0.336 | <0.001 | ­0.381 | <0.001 |

Data are expressed as partial correlation coefficient (r) adjusted for airway wall thickness of each generation bronchi and *p*-value. The other abbreviations are as described in Table S1.

Supplementary Table S4. Partial correlations between airway wall thickness and physiological function tests adjusted for airway luminal area in patients with COPD.

|  | Third-generation bronchi | | Fourth-generation bronchi | | Fifth-generation bronchi | | Sixth-generation bronchi | |
| --- | --- | --- | --- | --- | --- | --- | --- | --- |
|  | r | *p*-value | r | *p*-value | r | *p*-value | r | *p*-value |
| Spirometry |  |  |  |  |  |  |  |  |
| FVC, %predicted | 0.022 | 0.830 | 0.063 | 0.540 | 0.069 | 0.502 | 0.052 | 0.616 |
| FEV_1_, %predicted | ­-0.141 | 0.170 | ­-0.149 | 0.144 | -0.212 | 0.037 | -0.237 | 0.019 |
| FEV_1_/FVC ratio | ­-0.252 | 0.013 | -0.294 | 0.003 | -0.336 | <0.001 | -0.364 | <0.001 |
| MMF, %predicted | -0.154 | 0.132 | ­-0.186 | 0.068 | -0.234 | 0.021 | -0.275 | 0.006 |
| Respiratory impedance, whole-breath | | | | | | | | |
| R5 | 0.232 | 0.022 | 0.263 | 0.009 | 0.153 | 0.134 | 0.113 | 0.269 |
| R20 | 0.140 | 0.172 | 0.169 | 0.098 | 0.048 | 0.639 | -0.007 | 0.944 |
| R5-R20 | 0.343 | <0.001 | 0.363 | <0.001 | 0.321 | 0.001 | 0.298 | 0.003 |
| X5 | ­-0.191 | 0.060 | ­-0.187 | 0.067 | -0.138 | 0.178 | -0.132 | 0.198 |
| Fres | 0.240 | 0.018 | 0.280 | 0.005 | 0.209 | 0.040 | 0.231 | 0.023 |
| ALX | 0.252 | 0.013 | 0.272 | 0.007 | 0.182 | 0.074 | 0.192 | 0.060 |

Data are expressed as partial correlation coefficient (r) adjusted for airway luminal area of each generation bronchi and *p*-value. The other abbreviations are as described in Table S1.

Supplementary Table S5. Correlations between morphological changes of the lungs and physiological function tests in reference subjects.

|  | Ai | | | | | | | | WT | | | | | | | |
| --- | --- | --- | --- | --- | --- | --- | --- | --- | --- | --- | --- | --- | --- | --- | --- | --- |
|  | 3rd | | 4th | | 5th | | 6th | | 3rd | | 4th | | 5th | | 6th | |
|  | r | *p*-value | r | *p*-value | r | *p*-value | r | *p*-value | r | *p*-value | r | *p*-value | r | *p*-value | r | *p*-value |
| FVC, % | 0.002 | 0.990 | -0.076 | 0.606 | -0.129 | 0.384 | -0.140 | 0.349 | -0.029 | 0.843 | 0.059 | 0.688 | 0.025 | 0.865 | -0.026 | 0.862 |
| FEV_1_, % | -0.090 | 0.542 | -0.116 | 0.434 | -0.059 | 0.690 | -0.018 | 0.902 | -0.148 | 0.316 | 0.023 | 0.876 | 0.015 | 0.922 | 0.036 | 0.811 |
| FEV_1_/FVC | -0.115 | 0.438 | -0.046 | 0.758 | 0.078 | 0.597 | 0.205 | 0.167 | -0.141 | 0.341 | -0.026 | 0.860 | 0.066 | 0.654 | 0.096 | 0.522 |
| MMF, % | -0.070 | 0.636 | -0.018 | 0.903 | 0.045 | 0.760 | 0.157 | 0.291 | -0.021 | 0.886 | 0.128 | 0.387 | 0.193 | 0.189 | 0.285 | 0.052 |
| R5 | -0.175 | 0.235 | -0.090 | 0.544 | -0.054 | 0.717 | -0.146 | 0.327 | -0.067 | 0.652 | -0.006 | 0.969 | -0.194 | 0.186 | -0.065 | 0.662 |
| R20 | -0.256 | 0.079 | -0.164 | 0.265 | -0.125 | 0.399 | -0.202 | 0.174 | -0.154 | 0.296 | -0.088 | 0.554 | -0.243 | 0.096 | -0.176 | 0.236 |
| R5-R20 | 0.005 | 0.973 | 0.040 | 0.787 | 0.046 | 0.756 | -0.042 | 0.779 | 0.185 | 0.208 | 0.138 | 0.348 | 0.020 | 0.893 | 0.123 | 0.411 |
| X5 | 0.080 | 0.587 | 0.026 | 0.860 | -0.039 | 0.791 | 0.037 | 0.802 | -0.076 | 0.607 | -0.062 | 0.675 | -0.097 | 0.512 | -0.142 | 0.342 |
| Fres | 0.050 | 0.737 | 0.034 | 0.817 | 0.091 | 0.540 | -0.004 | 0.977 | 0.142 | 0.336 | 0.034 | 0.818 | 0.082 | 0.581 | 0.084 | 0.575 |
| ALX | 0.028 | 0.849 | 0.017 | 0.909 | 0.065 | 0.658 | -0.037 | 0.804 | 0.100 | 0.500 | 0.029 | 0.846 | 0.079 | 0.595 | 0.105 | 0.480 |

Data are expressed as Spearman rank correlation coefficient (r) and *p*-value. Ai, airway inner luminal area; WT, wall thickness. The other abbreviations are as described in Table S1.
